# A U-Shaped Relationship Between Selenium Concentrations and All-Cause or Cardiovascular Mortality in Patients With Hypertension

**DOI:** 10.3389/fcvm.2021.671618

**Published:** 2021-07-30

**Authors:** Qiu-hong Tan, Yu-qing Huang, Xiao-cong Liu, Lin Liu, Kenneth Lo, Ji-yan Chen, Ying-qing Feng

**Affiliations:** ^1^School of Biology and Biological Engineering, South China University of Technology, Guangzhou, China; ^2^Department of Cardiology, Guangdong Cardiovascular Institute, Guangdong Provincial People's Hospital, Guangdong Academy of Medical Sciences, Guangzhou, China; ^3^Department of Epidemiology, Centre for Global Cardio-metabolic Health, Brown University, Providence, RI, United States

**Keywords:** selenium, hypertension, all-cause mortality, cardiovascular mortality, risk factors

## Abstract

**Background:** Given the antioxidant activity of selenium, it has been reported benefits for blood pressure control and hypertension prevention, but few studies have investigated the association between serum selenium with mortality in hypertensive population.

**Methods:** All participants with hypertension aged ≥18 years at baseline were recruited from the National Health and Nutritional Examination Surveys (NHANES) 2003–2004, and followed for mortality through December 31, 2015. Subjects were categorized by quartiles of serum selenium (Q1: ≤124 μg/L, Q2: 125–135 μg/L, Q3: 136–147 μg/L, Q4: ≥148 μg/L). Multivariate Cox regression were implemented to estimate hazard ratios (HRs) and 95% confidence intervals (CIs). Restricted cubic spline analysis and two-piecewise linear regression were used to evaluate the relationship of serum selenium with mortality. Survival curves were used to depict cause-specific mortalities.

**Results:** A total of 929 participants (52.53% were male) were eligible for the current study with the average age of 63.10 ± 12.59 years. There were 307 deaths occurred including 56 cardiovascular death events during the mean follow-up time of 121.05 ± 40.85 months. A U-shaped association was observed between serum selenium and all-cause or cardiovascular mortality. In fully adjusted model, comparisons among quartiles revealed that risks of all-cause [HR (95%CI), 0.57 (0.39–0.81)] and cardiovascular death [HR (95%CI), 0.33 (0.13–0.86)] were lower in Q3. The nadir mortality of all-cause and cardiovascular was occurred at the serum selenium level of 136 μg/L and 130 μg/L, respectively.

**Conclusion:** Serum selenium concentration showed a U-shaped association with all-cause and cardiovascular mortality.

## Introduction

Selenium is one of the essential trace element for human beings that play a role via selenoproteins (1). It has for long been suggested as a protective factor for cancer, critical illness, diabetes, and cardiovascular disease through functions of antioxidant, anti-inflammation and reducing platelet aggregation (2, 3). Given oxidative stress is showed to be a primary contributor to hypertension development (4), sufficient selenium may benefit blood pressure control and hypertension prevention (5, 6). Deficient or excessive concentration of serum selenium has been reported closely correlated to elevated prevalence of hypertension (6–8), especially for subjects in the selenium-replete region (9). Because of the different contents of selenium in the environment, the concentration of selenium varies greatly worldwide. Compared with Europe, the United States demonstrated a higher level of selenium in soil and food intake (10). As a consequence, the average serum selenium concentration in most American adults was above 95 ng/mL (11), while Europeans ranged from 50 to 90 ng/mL (12).

Published literatures presented controversial conclusions of the efficacy of selenium concentration on mortality. Several studies reported a negative correlation between selenium status and risk of all-cause or cardiovascular death (13–15). However, the Third National Health and Nutrition Examination Survey (NHANES III; 1988–1994) reported that no association was found between serum selenium and cardiovascular mortality (16). Another prospective study conducted in China showed that no association was observed between serum selenium and overall mortality (17). To our knowledge, there was no investigation about the relationship of selenium status with mortality in hypertensive population. Thus, this study sought to evaluate the association between serum selenium concentration with all-cause and cardiovascular mortality in patients with hypertension.

## Materials and Methods

### Study Population

Data for the current study were abstracted from the NHANES database, which aimed at evaluating the health and nutritional status in the US population. It was conducted by the National Center for Health Statistics (NCHS) within the United States Centers for Disease Control and Prevention (CDC). The study survey was administrated through face-to-face interviews and physical examination in the mobile examination center (MEC), and the resulting data release by a 2-year cycle. Further information about NHANES was available on the website (https://www.cdc.gov/nchs/nhanes/index.htm).

**Figure 1:**
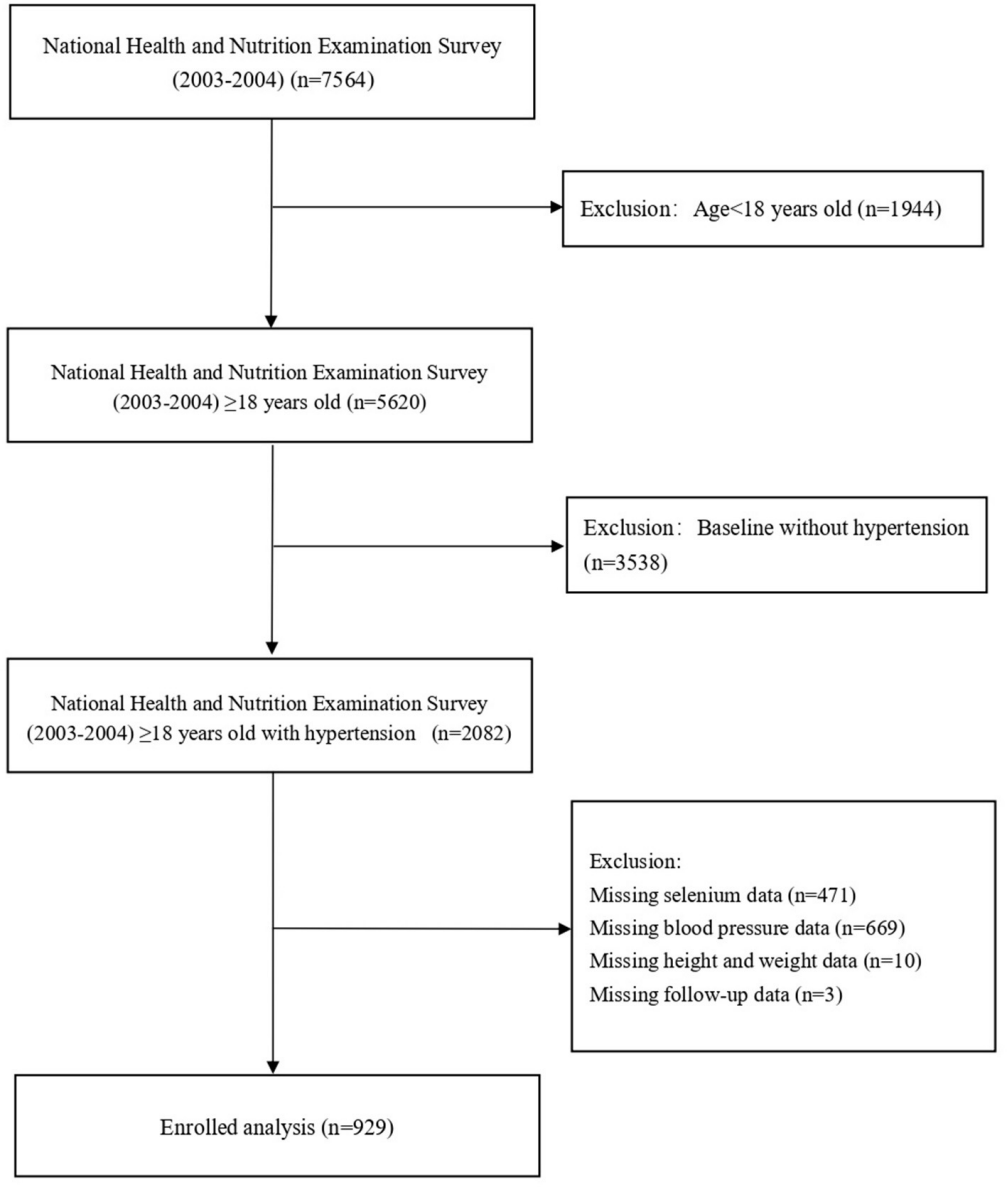
Study cohort.

In total, 7,564 participants were enrolled from NHANES 2003–2004. Subjects younger than 18 years of age (*n* = 1,944), without hypertension (*n* = 3,538), had missing data on selenium (*n* = 471), blood pressure (*n* = 669), height and weight (*n* = 10), or lost to follow-up (*n* = 3) were excluded. Thus, 929 participants were included in the final list (Figure 1). The survey was approved by the Institutional Review Board of the CDC (ethical approval code: Protocol #98-12). Signed informed consent was obtained from each subject.

### Assessment of Serum Selenium

Measurements of serum selenium was obtained at baseline. Blood drawn must be performing in the fasting state. After centrifugation, serum specimens should be stored at 4°C for transport and frozen at −20°C or at −70°C until time for analysis. Inductively Coupled Plasma-Dynamic Reaction Cell-Mass Spectrometry (ICP-DRC-MS) method was used to detect serum selenium concentration at the Trace Elements Laboratory, State of New York Department of Health, Wadsworth Center. More detail about the laboratory analyses procedures were described elsewhere (18).

### Covariate Data Collection

Information on sex, age, race, alcohol consumption, education level, smoking behavior, marital status, history of cardiovascular disease and cancer, medication history (including antihypertensive drugs, hypoglycemic agents, antiplatelet drugs and lipid-lowering drugs) were obtained through face-to-face questionnaire. Hypertension was defined as the examined systolic blood pressures (SBP) ≥ 130 mmHg or/and diastolic blood pressure (DBP) ≥ 80 mmHg (19), confirmed to be taking antihypertensive medications, or self-reported history of hypertension. Diabetes was defined as fasting blood glucose (FBG) ≥ 126 mg/dL, self-report, hemoglobin A1c(HbA1C) ≥6.5% (20), or using hypoglycaemic drugs. SBP, DBP, height and weight were measured in accordance with laboratory procedures in MEC. Body mass index (BMI) was calculated as weight (kilograms) divided by height (meters) squared (kg/m^2^) (21). Total cholesterol (TC, mg/dL), high-density lipoprotein cholesterol (HDL-C, mg/dL), and C-reactive protein (CRP, mg/dL) were assessed under standardized procedure and protocol. The estimated glomerular filtration rate (eGFR, mL/min/1.73 m^2^) was calculated using the Chronic Kidney Disease Epidemiology equation (22).

### Outcomes

The primary outcome of the current study was all-cause and cardiovascular mortality. Mortality data were obtained from the NHANES Public-use Linked Mortality Files through December 31, 2015, provided through the CDC. All-cause mortality was defined as death resulting from any cause. Cardiovascular mortality was considered when I00-I09, I11, I13, I20-I51, or I60-I69 were recorded as the underlying cause of death, based on the International Classification of Diseases (ICD-10) codes.

### Statistical Analysis

Participants were categorized by serum selenium quartiles (Q1: ≤124 μg/L, Q2: 125–135 μg/L, Q3: 136–147 μg/L, Q4: ≥148 μg/L). Descriptive data are expressed as mean ± standard deviation (SD) for continuous variables or as proportions for categorical variables. The one-way ANOVA and chi-square were used to assess differences among selenium quartiles or mortality and survival group. Cox proportional hazard regression models were performed to calculate hazard ratios (HRs) and 95% confidence intervals (CIs) for all-cause and cardiovascular mortality. Crude analysis adjust for none; adjusted model adjust for: age, sex, and race; fully adjusted model adjust for: age, sex, race, education level, marital status, smoking, BMI, SBP, TC, HDL-C, CRP, alcohol consumption, eGFR, comorbidities (cardiovascular disease, diabetes, and cancer), and medication use (lipid-lowering drugs, hypoglycemic agents, antiplatelet drugs, and antihypertensive drugs). Restricted cubic spline analysis was performed to examine the non-linearity of serum selenium and mortality. Three knots (10th, 50th, 90th percentiles of serum selenium distribution, corresponding to 115,135, and 159 μg/L, respectively) were used for restricted cubic spline modeling, with the lowest level of risk as the reference value. If a non-linear correlation was detected, a recursive algorithm was conducted to calculate the inflection point and a two-piecewise Cox proportional hazards model on both sides of the inflection point was then performed. Threshold level was defined by choosing the inflection point with maximum model likelihood, along with a log-likelihood ratio test to examine the statistical difference with one-line Cox proportional hazards model (23). Survival probability of serum selenium quartiles was presented by Kaplan–Meier curves and the log-rank test. All analyses were performed with R version 3.6.3 (R Foundation for Statistical Computing, Vienna, Austria).

## Results

### Baseline Demographic and Clinical Parameters

The current study enrolled 929 participants with mean age of 63.10 ± 12.59 years, 488 (52.53%) of them were males. The mean level of selenium was 136.78 ± 20.32 μg/L. Participants with higher selenium levels were more prone to be male, diabetics, having hypoglycemic agents, elevated TC, and reduced CRP. During the mean follow-up time of 121.05 ± 40.85 months, 307 (33.05%) deaths were recorded including 56 (6.03%) cardiovascular deaths (Table 1). Participants in survival group were on average younger in age, higher proportion being female, lower prevalence of smoking, diabetes, cancer, and cardiovascular disease, and had higher TC, DBP, eGFR, BMI, and education level, lower CRP level than participants in other two groups (cardiovascular death group and non-cardiovascular death group) (Table 2).

**Table 1:** Baseline demographic and clinical parameters among participants by selenium quartiles.

	**Total**	**Q1**	**Q2**	**Q3**	**Q4**	***P*-value**
Number	929	246	231	223	229	
Age, years	63.10 ± 12.59	62.33 ± 12.56	63.96 ± 13.09	62.37 ± 12.08	63.79 ± 12.58	0.331
Body mass index, kg/m^2^	29.79 ± 5.79	30.19 ± 6.67	30.17 ± 5.78	29.35 ± 5.00	29.43 ± 5.46	0.221
Systolic blood pressure, mmHg	127.56 ± 17.66	127.22 ± 18.78	126.08 ± 16.99	128.42 ± 18.60	128.60 ± 16.06	0.386
Diastolic blood pressure, mmHg	70.60 ± 16.37	69.91 ± 17.87	68.82 ± 16.95	72.30 ± 15.45	71.49 ± 14.78	0.099
Total cholesterol, mg/dL	203.18 ± 43.45	198.33 ± 49.27	197.28 ± 41.55	207.07 ± 38.65	210.58 ± 41.78	0.001
HDL cholesterol, mg/dL	52.69 ± 15.50	54.20 ± 16.20	51.45 ± 15.55	52.50 ± 14.91	52.50 ± 15.20	0.275
C-reactive protein, mg/L	0.77 ± 1.71	1.11 ± 2.55	0.70 ± 1.19	0.67 ± 1.33	0.58 ± 1.24	0.003
Alcohol consumption, gm	8.65 ± 27.02	8.51 ± 28.82	8.43 ± 29.72	9.76 ± 25.98	7.94 ± 23.00	0.910
Selenium, μg/L	136.78 ± 20.32	115.49 ± 7.40	129.96 ± 3.09	140.72 ± 3.46	162.68 ± 19.39	<0.001
eGFR, mg/min/1.73 m^2^	72.34 ± 20.40	73.20 ± 20.46	72.73 ± 21.53	71.94 ± 19.26	71.39 ± 20.30	0.779
Sex, *n* (%)						0.015
Male	488 (52.53)	108 (43.90)	125 (54.11)	124 (55.61)	131 (57.21)	
Female	441 (47.47)	138 (56.10)	106 (45.89)	99 (44.39)	98 (42.79)	
Education level, *n* (%)						0.582
Less than high school	306 (32.97)	84 (34.29)	80 (34.63)	75 (33.63)	67 (29.26)	
High school or above	622 (67.03)	161 (65.71)	151 (65.37)	148 (66.37)	162 (70.74)	
Marital status, *n* (%)						0.175
Married	380 (40.95)	115 (46.75)	91 (39.39)	83 (37.39)	91 (39.74)	
Other	548 (59.05)	131 (53.25)	140 (60.61)	139 (62.61)	138 (60.26)	
Smoking, *n* (%)						0.616
No	394 (42.41)	97 (39.43)	96 (41.56)	98 (43.95)	103 (44.98)	
Yes	535 (57.59)	149 (60.57)	135 (58.44)	125 (56.05)	126 (55.02)	
Race, *n* (%)						0.052
White	541 (58.23)	145 (58.94)	120 (51.95)	128 (57.40)	148 (64.63)	
Other races	388 (41.77)	101 (41.06)	111 (48.05)	95 (42.60)	81 (35.37)	
Diabetes, *n* (%)						0.007
No	673 (72.44)	196 (79.67)	166 (71.86)	161 (72.20)	150 (65.50)	
Yes	256 (27.56)	50 (20.33)	65 (28.14)	62 (27.80)	79 (34.50)	
Cardiovascular disease, *n* (%)						0.109
No	811 (88.25)	208 (85.60)	193 (85.78)	202 (90.99)	208 (90.83)	
Yes	108 (11.75)	35 (14.40)	32 (14.22)	20 (9.01)	21 (9.17)	
Cancer, *n* (%)						0.711
No	791 (85.51)	210 (86.42)	197 (85.28)	185 (83.33)	199 (86.90)	
Yes	134 (14.49)	33 (13.58)	34 (14.72)	37 (16.67)	30 (13.10)	
Antihypertensive drugs, *n* (%)						0.143
No	309 (33.26)	92 (37.40)	64 (27.71)	73 (32.74)	80 (34.93)	
Yes	620 (66.74)	154 (62.60)	167 (72.29)	150 (67.26)	149 (65.07)	
Lipid-lowering drugs, *n* (%)						0.821
No	679 (73.09)	183 (74.39)	167 (72.29)	166 (74.44)	163 (71.18)	
Yes	250 (26.91)	63 (25.61)	64 (27.71)	57 (25.56)	66 (28.82)	
Hypoglycemic agents, *n* (%)						0.005
No	777 (83.64)	219 (89.02)	194 (83.98)	188 (84.30)	176 (76.86)	
Yes	152 (16.36)	27 (10.98)	37 (16.02)	35 (15.70)	53 (23.14)	
Antiplatelet drugs, *n* (%)						0.645
No	893 (96.12)	238 (96.75)	219 (94.81)	216 (96.86)	220 (96.07)	
Yes	36 (3.88)	8 (3.25)	12 (5.19)	7 (3.14)	9 (3.93)	
Cardiovascular disease mortality, *n* (%)						0.034
No	873 (93.97)	227 (92.28)	221 (95.67)	216 (96.86)	209 (91.27)	
Yes	56 (6.03)	19 (7.72)	10 (4.33)	7 (3.14)	20 (8.73)	
All-cause mortality, *n* (%)						0.019
No	622 (66.95)	152 (61.79)	149 (64.50)	167 (74.89)	154 (67.25)	
Yes	307 (33.05)	94 (38.21)	82 (35.50)	56 (25.11)	75 (32.75)	

*N, number; HDL, high-density lipoprotein; eGFR, estimated glomerular filtration rate*.

*Values are mean ± standard deviation or n (%)*.

*P-value by one-way analysis of variance (ANOVA) test for continuous data and chi-square test for categorical variables among selenium quartiles*.

**Table 2:** Baseline demographic and clinical parameters among participants between mortality and survival group.

	**Survival**	**Total death**		***P*-value**
		**Cardiovascular death**	**Non-cardiovascular death**	
Number	622	56	251	
Age, years	58.91 ± 11.13	72.29 ± 11.55	71.45 ± 10.88	<0.001
Body mass index, kg/m^2^	30.18 ± 5.90	28.71 ± 5.95	29.08 ± 5.37	0.014
Systolic blood pressure, mmHg	127.99 ± 17.22	126.95 ± 21.66	126.63 ± 17.77	0.568
Diastolic blood pressure, mmHg	73.39 ± 15.68	64.14 ± 17.26	65.12 ± 16.12	<0.001
Total cholesterol, mg/dL	206.60 ± 41.45	205.43 ± 54.12	194.22 ± 44.54	<0.001
HDL cholesterol, mg/dL	53.16 ± 14.97	54.57 ± 15.38	51.09 ± 16.70	0.13
C-reactive protein, mg/L	0.67 ± 1.41	1.26 ± 3.59	0.93 ± 1.72	0.011
Alcohol consumption, gm	8.32 ± 22.01	6.51 ± 21.36	9.94 ± 37.37	0.61
Selenium, μg/L	137.08 ± 17.50	138.95 ± 28.73	135.54 ± 24.25	0.426
eGFR, mg/min/1.73 m^2^	76.79 ± 18.21	60.38 ± 18.82	63.95 ± 22.15	<0.001
Sex, *n* (%)				<0.001
Male	300 (48.23)	41 (73.21)	147 (58.57)	
Female	322 (51.77)	15 (26.79)	104 (41.43)	
Education level, *n* (%)				<0.001
Less than high school	180 (28.94)	26 (46.43)	100 (40.00)	
High school or above	442 (71.06)	30 (53.57)	150 (60.00)	
Marital status, *n* (%)				<0.001
Married	209 (33.66)	34 (60.71)	137 (54.58)	
Other	412 (66.34)	22 (39.29)	114 (45.42)	
Smoking, *n* (%)				0.002
No	286 (45.98)	14 (25.00)	94 (37.45)	
Yes	336 (54.02)	42 (75.00)	157 (62.55)	
Race, *n* (%)				<0.001
White	334 (53.70)	41 (73.21)	166 (66.14)	
Other races	288 (46.30)	15 (26.79)	85 (33.86)	
Diabetes, *n* (%)				<0.001
No	478 (76.85)	33 (58.93)	162 (64.54)	
Yes	144 (23.15)	23 (41.07)	89 (35.46)	
Cardiovascular disease, *n* (%)				<0.001
No	566 (91.44)	39 (72.22)	206 (83.74)	
Yes	53 (8.56)	15 (27.78)	40 (16.26)	
Cancer, *n* (%)				<0.001
No	550 (88.71)	46 (82.14)	195 (78.31)	
Yes	70 (11.29)	10 (17.86)	54 (21.69)	
Antihypertensive drugs, *n* (%)			<0.001
No	235 (37.78)	15 (26.79)	59 (23.51)	
Yes	387 (62.22)	41 (73.21)	192 (76.49)	
Lipid-lowering drugs, *n* (%)				0.07
No	469 (75.40)	37 (66.07)	173 (68.92)	
Yes	153 (24.60)	19 (33.93)	78 (31.08)	
Hypoglycemic agents, *n* (%)				0.004
No	538 (86.50)	44 (78.57)	195 (77.69)	
Yes	84 (13.50)	12 (21.43)	56 (22.31)	
Antiplatelet drugs, *n* (%)				<0.001
No	604 (97.11)	48 (85.71)	241 (96.02)	
Yes	18 (2.89)	8 (14.29)	10 (3.98)	

*N, number; HDL, high-density lipoprotein; eGFR, estimated glomerular filtration rate*.

*Values are mean ± standard deviation or n (%)*.

*P-value: One-way analysis of variance (ANOVA) test for continuous data and chi-square test for categorical variables*.

### Association of Serum Selenium With All-Cause and Cardiovascular Mortality

The results of the Cox regression analysis were listed in Table 3. Taking the lowest quartiles (Q1) as reference, the full-adjusted HRs for all-cause mortality among groups (Q2, Q3, and Q4) were 0.74 (95%CI, 0.54–1.03), 0.57 (95%CI, 0.39–0.81), and 0.74 (95%CI, 0.53–1.04), respectively. For cardiovascular mortality, these numbers were 0.38 (95%CI, 0.16–0.90), 0.33 (95%CI, 0.13–0.86), and 0.94 (95%CI, 0.46–1.90), respectively. Thus, risk for all-cause or cardiovascular death by categorized selenium level suggesting a U-shaped association, which was lowest in Q3, and the beneficial effects could be attenuated in Q4.

**Table 3:** The association of selenium levels with all-cause mortality and cardiovascular mortality.

	**Crude analysis** **HR (95%CI) *P*-value**	**Adjusted model** **HR (95%CI) *P*-value**	**Fully adjusted model** **HR (95%CI) *P*-value**
**All-cause mortality**			
Selenium per 10 μg/L increment	0.98 (0.92, 1.04) 0.4502	0.95 (0.90, 1.01) 0.0842	0.96 (0.90, 1.03) 0.2486
Selenium quartiles			
Q1	1.0	1.0	1.0
Q2	0.88 (0.65, 1.18) 0.3826	0.75 (0.56, 1.01) 0.0555	0.74 (0.54, 1.03) 0.0715
Q3	0.60 (0.43, 0.83) 0.0024	0.56 (0.40, 0.78) 0.0006	0.57 (0.39, 0.81) 0.0020
Q4	0.83 (0.61, 1.12) 0.2238	0.72 (0.53, 0.97) 0.0333	0.74 (0.53, 1.04) 0.0802
*p* for trend	0.0574	0.0110	0.0419
**Cardiovascular disease mortality**			
Selenium per 10 μg/L increment	1.05 (0.93, 1.18) 0.4572	1.01 (0.90, 1.14) 0.8215	1.03 (0.90, 1.17) 0.6882
Selenium quartiles			
Q1	1.0	1.0	1.0
Q2	0.53 (0.25, 1.14) 0.1046	0.42 (0.20, 0.91) 0.0279	0.38 (0.16, 0.90) 0.0276
Q3	0.38 (0.16, 0.90) 0.0281	0.35 (0.15, 0.82) 0.0166	0.33 (0.13, 0.86) 0.0226
Q4	1.10 (0.58, 2.05) 0.7761	0.94 (0.50, 1.76) 0.8500	0.94 (0.46, 1.90) 0.8582
*p* for trend	0.9070	0.8987	0.9652

*HR, hazard ratio; CI, confidence interval. Ref, reference. Crude analysis adjust for none; Adjusted model adjust for: age, sex, and race; Fully adjusted model adjust for: age, sex, race, education level, marital status, smoking, body mass index, systolic blood pressure, total cholesterol, high density lipoprotein cholesterol, C-reactive protein, alcohol consumption, estimated glomerular filtration rate, comorbidities (cardiovascular disease, diabetes, and cancer), and medication use (lipid-lowering drugs, hypoglycemic agents, antiplatelet drugs, and antihypertensive drugs)*.

*P-value by Cox proportional hazard regression models*.

### The Analyses of U-Shaped Relationship

**Figure 2:**
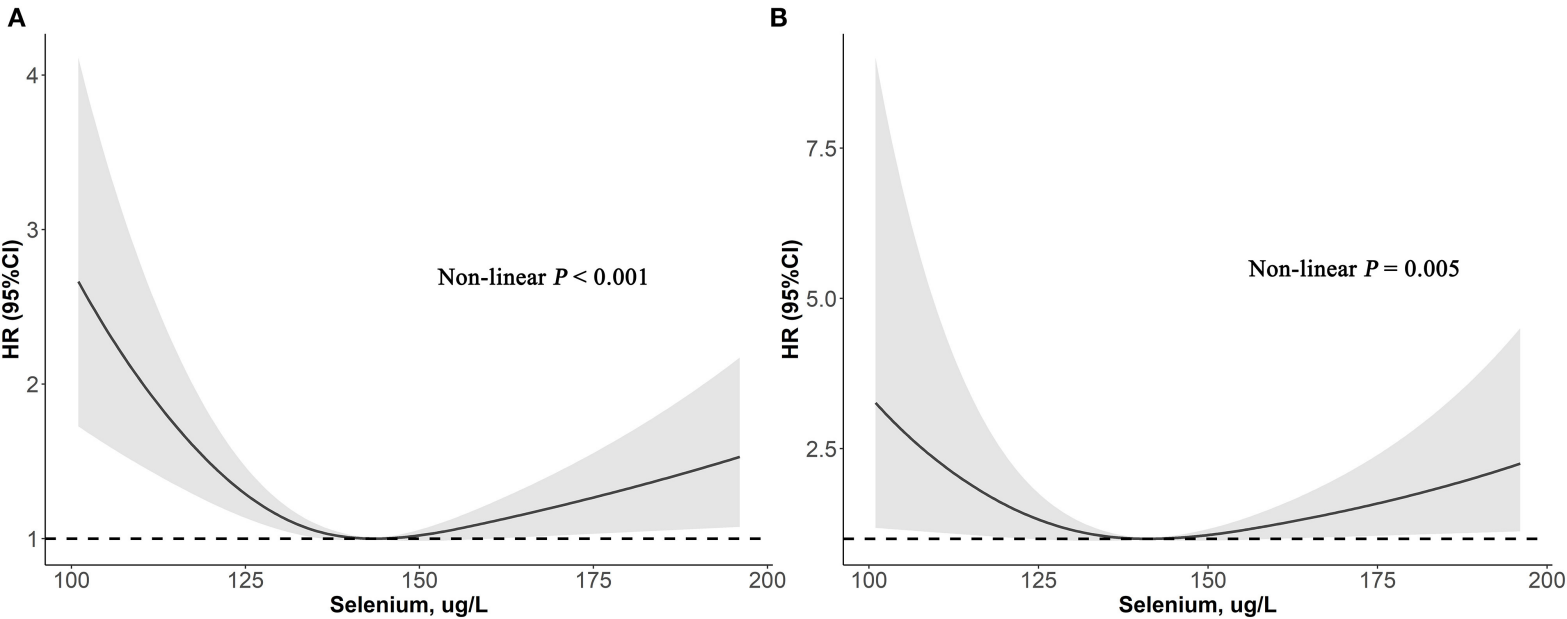
Association of selenium levels with the all-cause **(A)** and cardiovascular mortality **(B)** performed by restricted cubic spline analysis.

As depicted in Figure 2, results of restricted cubic spline regression found a U-shaped relationship between serum selenium with all-cause and cardiovascular mortality (Non-linear *p* < 0.001 and Non-linear *p* = 0.005, respectively). Furthermore, the two-piecewise linear regression revealed that the risk of all-cause mortality decreased up to a minimum at the selenium level of 136 μg/L, and then increased with higher selenium level [HR (95%CI): 0.75 (0.66–0.85), 1.08 (1.01–1.16) per 10 μg/L selenium increment in less or more than 136 μg/L, respectively]. Similarly, risk of cardiovascular death fell with rising serum selenium up to 130 μg/L, then ascended with greater level of selenium [HR (95%CI): 0.60 (0.42–0.86), 1.15 (1.02–1.31) per 10 μg/L selenium increased in less or more than 130 μg/L, respectively] (Table 4). Therefore, a U-shaped association between serum selenium and all-cause or cardiovascular mortality was noted, and the nadir mortality was occurred at the serum selenium level of 136 and 130 μg/L, respectively.

**Table 4:** The results of two-piecewise linear regression model between selenium levels and all-cause or cardiovascular mortality.

	**All-cause mortality** **HR (95% CI) *P*-value**	**Cardiovascular mortality** **HR (95% CI) *P*-value**
Cutoff value, μg/L	136	130
<Cut-off value (per 10 μg/L increment)	0.75 (0.66, 0.85) <0.0001	0.60 (0.42, 0.86) 0.0051
>Cut-off value (per 10 μg/L increment)	1.08 (1.01, 1.16) 0.0207	1.15 (1.02, 1.31) 0.0255
P for log likelihood ratio test	<0.001	0.004

*HR, hazard ratio; CI, confidence interval*.

*Two-piecewise linear regression model were adjusted for age, sex, race, education level, marital status, smoking, body mass index, systolic blood pressure, total cholesterol, high density lipoprotein cholesterol, C-reactive protein, alcohol consumption, estimated glomerular filtration rate, comorbidities (cardiovascular disease, diabetes, and cancer), and medication use (lipid-lowering drugs, hypoglycemic agents, antiplatelet drugs, and antihypertensive drugs)*.

*P-value by two-piecewise Cox proportional hazards model*.

### Survival Analysis

**Figure 3:**
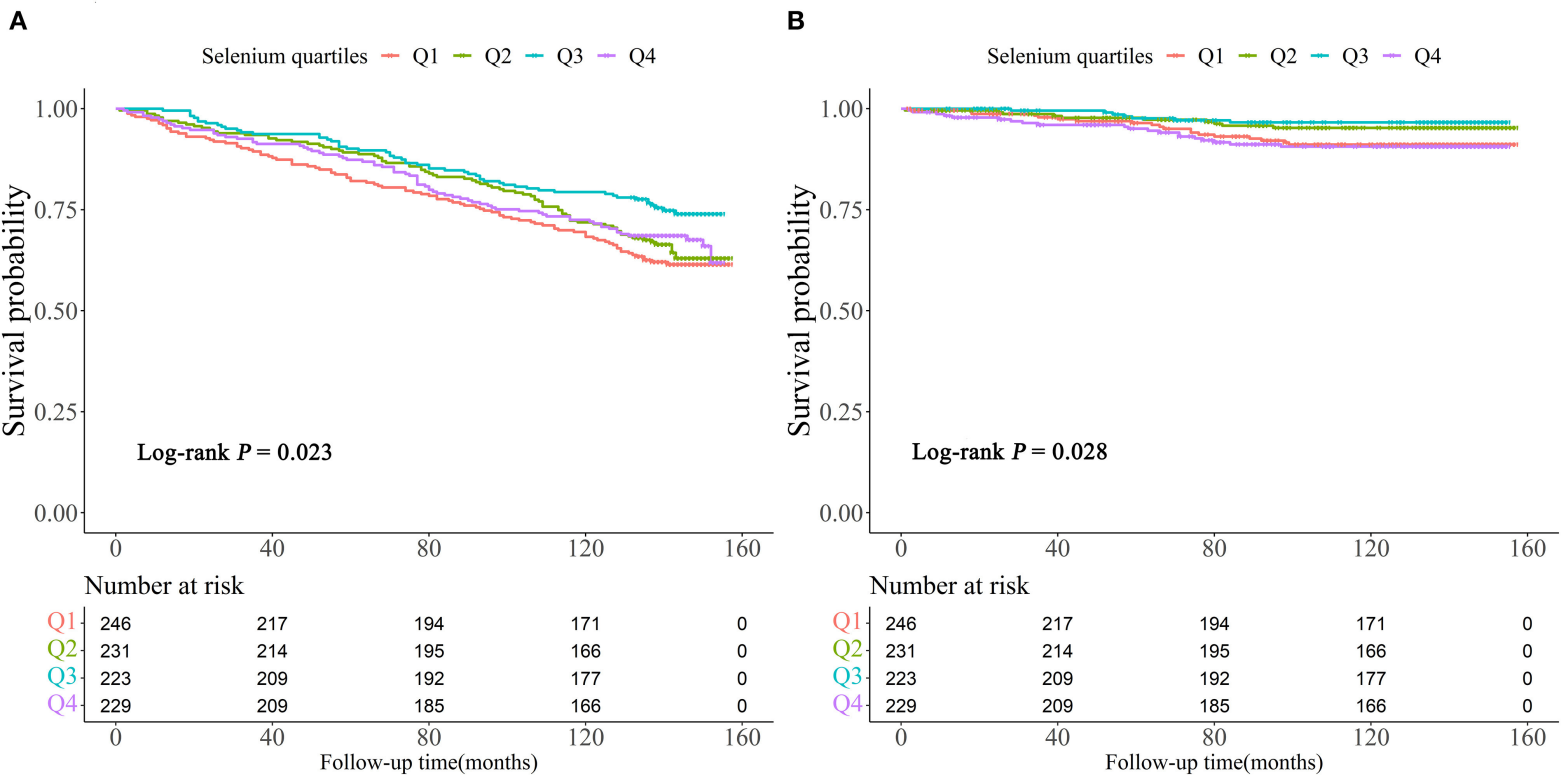
Kaplan-Meier survival curve for all-cause **(A)** and cardiovascular mortality **(B)**.

Kaplan-Meier survival curves of all patients stratified by selenium were demonstrated in Figure 3. It showed that participants in Q3 had the lowest risk of all-cause or cardiovascular mortality (Log-rank *P* = 0.023 and Log-rank *P* = 0.028, respectively).

## Discussion

This study found that the higher the selenium concentration, it more likely to accompanied by higher TC, and diabetes. Meanwhile, a U-shaped relationship between serum selenium with all-cause and cardiovascular mortality was observed in patients with hypertension, and the optimal cut-off value was 136 and 130 μg/L, respectively.

It has been showed that the relationship between selenium and death has been draw different conclusions in different populations. The AtheroGene study (24) followed up 879 participants with acute coronary syndrome during 6.1 years of median follow-up in Koblenz, Germany. In their research, the HR (95%CI) was 0.38 (0.16–0.91) for cardiovascular mortality comparing the highest (>84.4 μg/L) with the lowest (<64.0 μg/L) tertile of serum selenium, demonstrated a negative trend across the tertiles. Another prospective study of 347 community-dwelling older conducted in Italy found that after 10 years of follow-up time, compared with low serum selenium (≤105.3 μg/L), participants with high serum selenium (>105.3 μg/L) had a lower risk of all-cause death [HR(95%), 0.71(0.54–0.92)] (13). Interestingly, the two studies mentioned above were conducted in Europe, where the selenium intake and average serum selenium levels were lower than the United States (11, 12). Thus, their results for the lower level of selenium related to a higher risk of all-cause or cardiovascular death could compatible with our findings. However, a cohort study followed up with 1,103 adults during 15 years in China showed that no association was observed between serum selenium and total death (17), which was inconsistent with our finding. The reason for discrepancy may due to different adjustment factors. The previous study only adjusted for sex, age, smoking, drinking, and serum cholesterol, whereas our study adjusted for more potential confounding variables, such as anthropometric and demographic features, CRP, eGFR, comorbidities, and medication use.

The results of our study showed that too low and too high level of serum selenium may be linked to elevated all-cause and cardiovascular mortality were partially consistent with several previous research. The NHANES III (1988–1994) study (16) found that a U-shaped relationship was confirmed between serum selenium and all-cause mortality, instead, no association was observed between serum selenium and cardiovascular mortality. This discrepancy could be explained by the different characteristics of study population and detection methods. In the present research, the study population comprised people with hypertension were found to be older than the previous study involved a general population. Moreover, the ICP-DRC-MS method was used to assay selenium in our research, which reaching a high degree of precision demonstrated by a lower inter-assay coefficient of variation than the traditional method in the previous research. Li et al. (25) reported that the non-linear association was found between serum selenium and risks of all-cause death, however, the relationship between serum selenium with cardiovascular mortality was markedly in females only. The disparate results could be explained by the application of different versions and the range of ICD code, which may impact the recording of cardiovascular mortality. Death from cardiovascular disease was determined by ICD-10 (version presently applied, I00-I09, I11, I13, I20-I51, or I60-I69) in the current study, whereas previous studies used a broader set of ICD codes to define cardiovascular mortality, which may overestimate the prevalence of cardiovascular death.

Several potential mechanisms underlying our findings explained below. During the hypertensive state, bioavailability of antioxidants decreases and excessive reactive oxygen species (ROS) production eventually led to oxidative stress, cellular and tissue damage (26). Selenium is known to defense against oxidative stress through selenoproteins [including glutathione peroxidases (GPx) and thioredoxin reductases (TrxR)] (27). Low level of selenium could be limiting the synthesis of selenoproteins, leading to blunted effect of antioxidant and anti-inflammatory (11) and resulting in elevated risk of death (28). Nevertheless, with increasing selenium concentration, the GPx activity increases directly until reaching a plateau (29), and depletion of selenoproteins may occur when selenium oversupply resulting in health problem (30). In animal model, wistar rats with selenium supplement for 85 days showed elevation of blood pressure, indicating that longer-term selenium supplement may affect cardiovascular health (31). Furthermore, excessive exposure of selenium may also relate to harmful effects (32), including preventing proper protein folding (33), inducing the unfolded protein response (UPR) (34), causing production of superoxide and angiogenesis damage (30, 35).

The strengths of our study are the large sample size from a nationally representative population with strict adherence to protocol, and the use of a national register for identification of deaths. However, some limitations still exist. First, cardiovascular mortality is a complex phenomenon, and although we have adjusted for most relevant confounders, we cannot rule out residual or unknown confounding factors. Second, the exclusion of these patients who had missing data on selenium, blood pressure, height and weight may lead to selection bias. Third, serum selenium was only measured at baseline, and selenium exposure may have changed over time, which might have resulted in misclassification. Forth, some factors may affect selenium metabolism including supplement intake, living environment and dietary behaviors were not collected in our study.

In conclusion, serum selenium concentrations had a U-shaped relationship with all-cause and cardiovascular mortality in patients with hypertension, and the cut-off values were showed to be 136 and 130 μg/L, respectively. This suggested that too low or too high concentration of serum selenium might be concomitant with poor outcomes. The exact reaction mechanism of selenium in human in biological systems require further study.

## Data Availability Statement

Publicly available datasets were analyzed in this study. This data can be found here: https://www.cdc.gov/nchs/nhanes/index.htm.

## Ethics Statement

The studies involving human participants were reviewed and approved by Institutional Review Board of the Centers for Disease Control and Prevention. The patients/participants provided their written informed consent to participate in this study.

## Author Contributions

Y-qF, Y-qH, KL, and Q-hT: conceptualization and methodology. Y-qH, Q-hT, LL, and X-cL: formal analysis. Y-qH, J-yC, and Y-qF: supervision and validation. Q-hT and KL: writing and revision. All authors contributed to the article and approved the submitted version.

## Conflict of Interest

The authors declare that the research was conducted in the absence of any commercial or financial relationships that could be construed as a potential conflict of interest.

## Publisher's Note

All claims expressed in this article are solely those of the authors and do not necessarily represent those of their affiliated organizations, or those of the publisher, the editors and the reviewers. Any product that may be evaluated in this article, or claim that may be made by its manufacturer, is not guaranteed or endorsed by the publisher.
